# Novel hemizygous *CORO1A* variant leads to combined immunodeficiency with defective platelet calcium signaling and cell mobility

**DOI:** 10.1016/j.jacig.2023.100172

**Published:** 2023-09-27

**Authors:** Anna Khoreva, Kirill R. Butov, Elena I. Nikolaeva, Alexey Martyanov, Elena Kulakovskaya, Dmitry Pershin, Maxim Alexenko, Maria Kurnikova, Ruslan Abasov, Elena Raykina, Dmitry Abramov, Kristina Arnaudova, Yulia Rodina, Natalia Trubina, Yulia Skvortsova, Dmitry Balashov, Anastasia Sveshnikova, Alexey Maschan, Galina Novichkova, Mikhail Panteleev, Anna Shcherbina

**Affiliations:** a Dmitry Rogachev National Medical Center of Pediatric Hematology, Oncology and Immunology, Moscow, Russia; b Center for Theoretical Problems of Physicochemical Pharmacology, Moscow, Russia; c Faculty of Physics, Lomonosov Moscow State University, Moscow, Russia; d Astrakhan State Medical University, Astrakhan, Russia

**Keywords:** CORO1A, combined immunodeficiency, cytoskeleton, actin, granulomatous dermatitis, platelet

## Abstract

**Background:**

To date, fewer than 20 patients have been identified as having germline biallelic mutations in the coronin-1A gene (*CORO1A*) and its protein with clinical features of combined immunodeficiency characterized by T-cell lymphopenia ranging from the severe phenotype to the mild phenotype, recurrent infections, and lymphoproliferative disorders. However, the effects of CORO1A protein disruption on actin-dependent functions in primary cells have not been fully delineated.

**Objective:**

We sought to characterize the underlying defects of actin-dependent cellular functions in a female patient with combined immunodeficiency caused by a novel missense variant in the *CORO1A* gene in combination with a *de novo* heterozygous microdeletion of chromosome 16p11.2 and also to provide evidence of the pathogenicity of this gene mutation.

**Methods:**

To identify the genetic defect, next-generation sequencing followed by Sanger confirmation and array comparative genomic hybridization were performed. Western blot and quantitative PCR tests were used to assess the effects on the protein. Flow cytometry and live microscopy were performed to investigate cellular motility and immune cell counts and function.

**Results:**

We demonstrated that the *CORO1A* hemizygous variant c.19C>T, p. A7C induces significant decreases in cellular levels of the CORO1A protein while leaving mRNA concentrations unaffected. The observed mutation resulted in impaired natural killer cell cytotoxicity and platelet calcium signaling. In addition, primary granulocytes and mesenchymal cells showed significant defects in motility.

**Conclusion:**

Collectively, we added new data about the *CORO1A* gene as a key player in actin cytoskeleton dynamics and cell signaling. Our findings expand the clinical spectrum regarding CORO1A protein deficiency and confirm the importance of a personalized therapeutic approach for each patient.

## Introduction

Severe combined immunodeficiency (SCID) is a group of life-threatening disorders for which an increasing number of causative genetic defects have been described. This list has recently been enriched with the syndrome caused by CORO1A biallelic mutations. Since the first patient with this T^−^B^+^NK^+^ SCID was identified in 2009,^1^ a series of patients with CORO1A protein deficiency have been reported and the disease’s spectrum has been expanded to include a milder phenotype that is caused by hypomorphic *CORO1A* mutations and leads to T-cell lymphopenia of variable severity, profound defects of naive T-cells, seemingly preserved B- and natural killer (NK) cell compartments, recurrent viral infections, and EBV-associated B-cell lymphoproliferation.^2^, ^3^, ^4^, ^5^, ^6^, ^7^, ^8^, ^9^

The CORO1A gene is located on the 16p11.2 chromosome; it encodes eponymous protein belonging to the coronin family.^10^ CORO1A protein is predominantly expressed in hematopoietic cells and accumulates at the sites of cytoskeletal rearrangements.^11^ Together with a plethora of proteins, it is crucial to processes associated with F-actin and cytoskeletal dependence, such as phagocytosis, cell polarization, and migration.^12^ In contrast to the proteins that promote actin polymerization and branching, such as Wiskott-Aldrich syndrome protein, coronins inhibit the actin-related protein 2/3 (Arp2/3) by preventing it from binding to F-actin. Studies using *CORO1A* knockout mice demonstrated that this molecule is an essential regulator of T-cells’ actin dynamics, homeostasis, and trafficking and that its absence leads to a calcium-signaling defect downstream from the TCR receptor and stunted T-lymphocyte egress from the thymus.^13^

CORO1A has been investigated predominantly in lymphocytes, whereas in other hematopoietic cells—platelets, neutrophils, and macrophages—its role remains less well delineated. Recent studies in mouse models showed that the absence of CORO1A affects platelets’ spreading, aggregation, and thrombus formation,^14^^,^^15^ as well as neutrophil adhesion and postadhesion events.^16^

Herein, we describe a female patient with a novel missense mutation in the *CORO1A* gene and a *de novo* heterozygous microdeletion of the 16p11.2 chromosome, leading to combined immunodeficiency and underlying defects in cell motility and calcium signaling.

## Results and discussion

**Fig 1:**
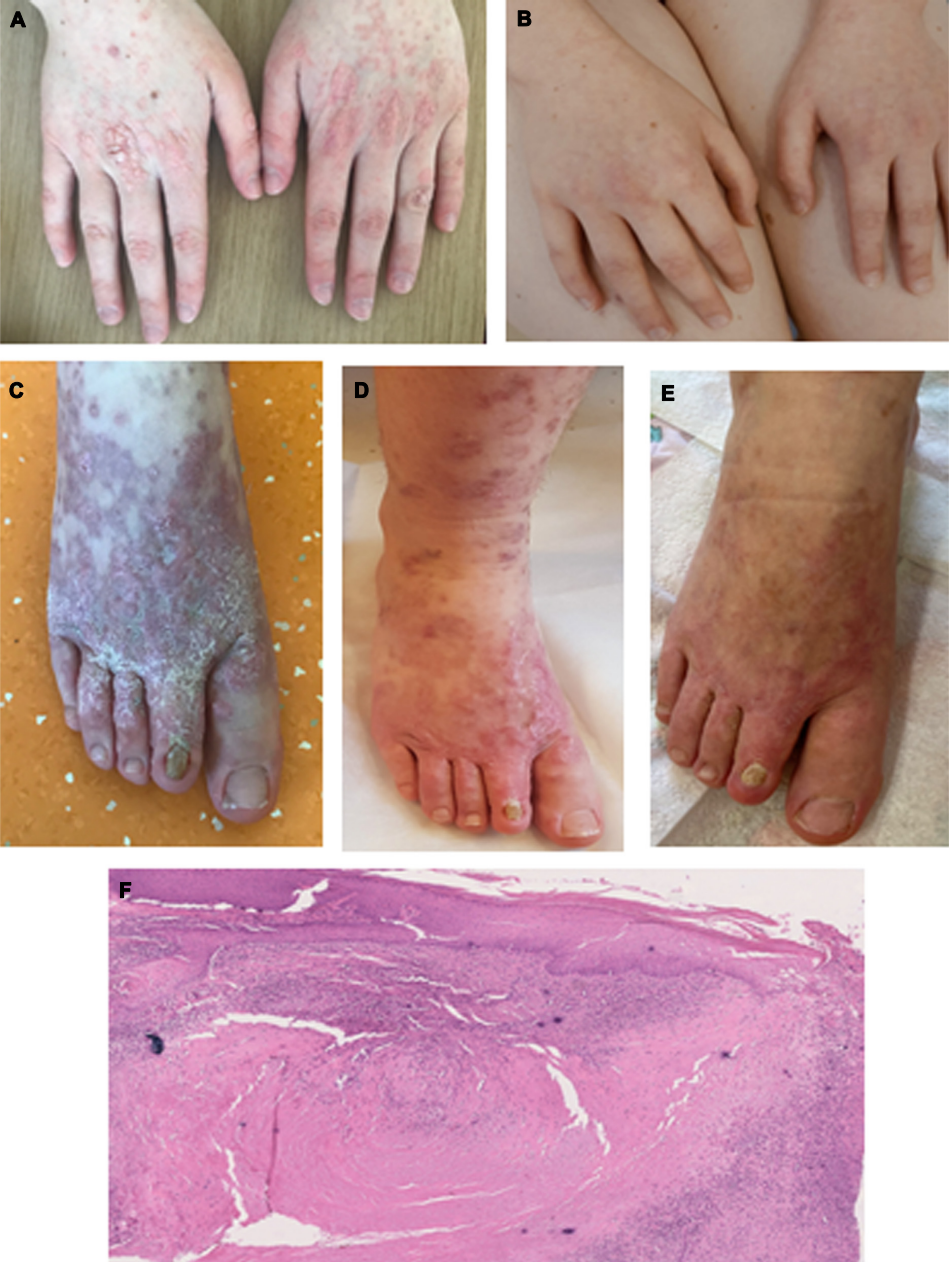
Dermatologic symptoms of the patient with CORO1A deficiency before and after treatment. **A** and **B,** Disseminated cutaneous papillomas before and 12 months after stem cell transplantation**. C-E,** Skin lesions before treatment (*left*), after 7 infusions (fewer inflammatory skin lesions) of infliximab, and 12 months after stem cell transplantation (*right*)**. F,** Skin lesion histology, demonstrating hyperkeratosis and spongiosis. Polygonal necrotizing granuloma is present in the dermis, surrounded by lymphocytes and histiocytes. Hematoxylin and eosin staining. Original magnification × 10.

The female patient was born to nonconsanguineous healthy parents. Her early physical and cognitive development was normal. Beginning at age 6 years, she experienced recurrent respiratory tract infections—including 3 cases of pneumonia-chronic polypoid sinusitis and severe chickenpox that required hospitalization. At age 12 years, she developed disseminated papillomatous lesions (hands and anogenital) and progressive ulcerating dermatitis on 1 foot and leg, which were resistant to systemic and topical antibiotics and steroids (Fig 1, *A-E*). Immunologic evaluation demonstrated low IgG levels, and intravenous immunoglobulin substitution was initiated. This reduced the number of respiratory tract infections but did not improve the patient’s skin disease. She was also diagnosed with autism spectrum disorder.

Following the patient’s evaluation at our center at age 15 years, her immunologic workup showed marked, predominantly T-cell lymphopenia (CD3^+^ cells, 0.69 × 10^9^/L) with low mitogen responses, absence of CD4^+^CD45RA^+^ naive T-cells, normal CD4^+^ and CD8^+^ effector memory cells levels, and increased CD3/TCRab^+^CD4^–^CD8^–^ cell levels (17.8%) (Table I).

**Table I:** Immunologic profile of the patient with COROA1 deficiency at the ages of 15 and 16 years and 1.5 years after stem cell transplantation

Parameter	At age 15 y	At age 16 y	At age 17 y (after HSCT)	Reference range
Serum immunoglobulin level (g/L)				
IgG	11.9∗	11.9∗	8.08	7-16
IgM	1.32	1.27	0.474	0.6-2.6
IgA	1.7	1.57	0.783	1-2.3
Lymphocyte subset analysis				
Lymphocyte count (× 10^9^/L)	1.27	1.25	2.54	1.5–3.9
T cells				
CD3^+^ (× 10^9^/L)	0.69	0.83	1.7	1.00-2.2
CD3^+^CD4^+^ (× 10^9^/L)	0.18	0.3	1.0	0.53-1.3
CD3^+^CD8^+^ (× 10^9^/L)	0.33	0.31	0.6	0.33-0.92
CD4^+^CD45RA^+^CD197^+^ naive T cells	0.001	0.002	0.7	0.23-0.77
CD4^+^CD45RA^–^CD197^–^ effector memory cells	0.111	0.277	0.19	0.1-0.6
CD4^+^CD45RA^–^CD197^+^ memory T cells	0.076	0.017	0.06	0.22-0.66
CD8^+^CD45RA^+^CD197^+^ naive T cells	0.002	0.004	0.28	0.24-0.71
CD8^+^CD45RA^–^CD197^–^ effector memory cells	0.207	0.223	0.23	0.1-0.6
CD8^+^CD45RA^–^CD197^+^ memory T cells	0.021	0.005	0.007	0.09-0.44
CD3/TCRab^+^CD4^–^CD8^–^ (% from CD3)	15	17.8		
CD3/TCRab^–^CD4^–^CD8^–^	0.0972	0.15		
B cells				
CD19^+^ (× 10^9^/L)	0.127	0.16	0.25	0.1-0.6
CD19^+^CD27^–^IgD^+^ naive (%)	61	54	77,23	51.3-82.5
CD19^+^CD27^–^IgD^+^ naive (× 10^9^/L)	0.078	0.087	0.195	0.12-0.43
CD19^+^CD27^+^IgD^+^ before switch of memory B cells (%)	6.3	6.8	0.36	4.6-18.2
CD19^+^CD27^+^IgD^+^ before switch of memory B cells (× 10^9^/L)	0.008	0.011	0.001	0.02-0.07
CD19^+^CD27^+^IgD^–^ after switch of memory B cells (%)	18.8	21	1.51	8.7-25.6
CD19^+^CD27^+^IgD^–^ after switch of memory B cells (× 10^9^/L)	0.024	0.034	0.004	0.03–0.11
NK cells				
CD3^–^CD16^+^CD56^+^ (× 10^9^/L)	0.4	0.24	0.59	0.07-0.48
Proliferation assay results (PHA)	Diminished			
Proliferation assay results (ConA)	Diminished			

*ConA*, Concanavalin A; *PHA*, phytohemagglutinin.

∗ Igg serum concentration on intravenous immunoglobulin substitution.

Immunohistologic analysis of the patient’s skin lesion biopsy specimen showed granulomas with central necrosis and cellular infiltrate comprising CD4^+^ and CD8^+^ lymphocytes, as well as CD163^+^ and CD68^+^ cells (Fig 1, *F*). A tissue sample was negative for bacteria, fungi (culture), herpetic and rubella viruses (PCR), and *Mycobacterium* spp (including nontuberculous Mycobacteria and *Mycobacterium leprae* [PCR]).

In an attempt to treat the patient’s skin granuloma, she was given the anti–TNF-α preparation (infliximab, 5 mg/kg, at weeks 0, 2, 4, 6 and then every 8 weeks), with only slight improvement of the lesion (Fig 1, *C* and *D*).

**Fig 2:**
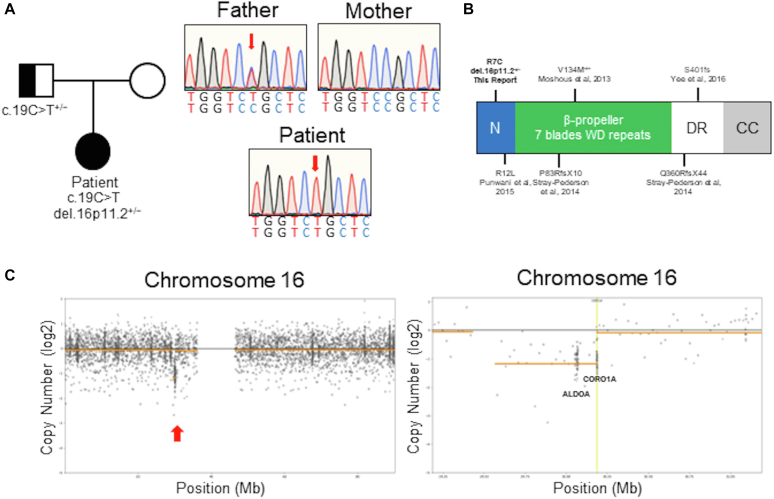
Pedigree and mutation analysis of the patient with CORO1A deficiency**. A,** Pedigree of the patient and Sanger sequencing demonstrating *CORO1A* mutations in a hemizygous state in the patient and in a heterozygous state in her father. **B,** CORO1A structure with previously published and current missense mutations locations. **C,** Representative plots of chromosome 16 next-generation sequencing gene panel coverage showing decreases in *CORO1A* and *ALDOA* coverage.

Next-generation sequencing using a custom gene panel revealed the novel hemizygous *CORO1A* (NM_007074.3) variant c.19C>T, p.Arg7Cys, which was subsequently confirmed by using Sanger sequencing. The patient’s father was a heterozygous carrier of the variant. The patient’s mother’s DNA sequence was determined to be of the wild type (Fig 2, *A*). The mutation was not found in the dbSNP, HGMD, or gnomAD databases, and it was predicted to be damaging or probably damaging by most *in silico* algorithms (eg, SIFT, PolyPhen, PROVEAN). Further analysis revealed a 2-fold coverage decrease in the *CORO1A* and *ALDOA* genes (Fig 2, *C*). Copy number analysis of the patient’s DNA using an exon-level oligonucleotide array (array comparative genomic hybridization) revealed a decrease in probe intensity in the 576-kb area on the short arm of chromosome 16, indicating a large heterozygous deletion (arr[hg38] 16p11.2(29613185_30189223) × 1), which included the whole *CORO1A* gene, as well as 26 other genes (see the Supplementary Methods in the Online Repository at www.jaci-global.org). Array comparative genomic hybridization did not detect any deletions in the patient’s mother’s DNA, thus confirming the *de novo* origin.

At age 16 years, the patient underwent haploidentical hematopoietic stem cell transplantation (HSCT) with TCRαβ/CD19 depletion from her unaffected mother.^17^ The conditioning regimen consisted of treosulfan (42 g/m^2^), fludarabine (150 mg/m^2^), melphalan (140 mg/m^2^), horse antithymocyte globulin (100 mg/kg), rituximab (200 mg), abatacept (10 mg/kg), tocilizumab (8 mg/kg), and bortezomib (1.3 mg/m^2^) without posttransplant graft-versus-host disease prophylaxis. Neutrophil engraftment was detected on day 17 after HSCT and platelets engraftment on day 20 after transplantation. Three months after HSCT, the patient developed acute grade 2 skin graft-versus-host disease, which responded fully to treatment with cyclosporine A. The cyclosporine A treatment was discontinued after 7 months. The patient is currently at 12 months after HSCT, without immunosuppression and with full donor chimerism and good immune function (Table I); in addition, she is no longer undergoing immunoglobulin substitution. Her granulomatous skin lesion gradually improved, and her papillomas resolved (Fig 1, *B* and *E*).

**Fig 3:**
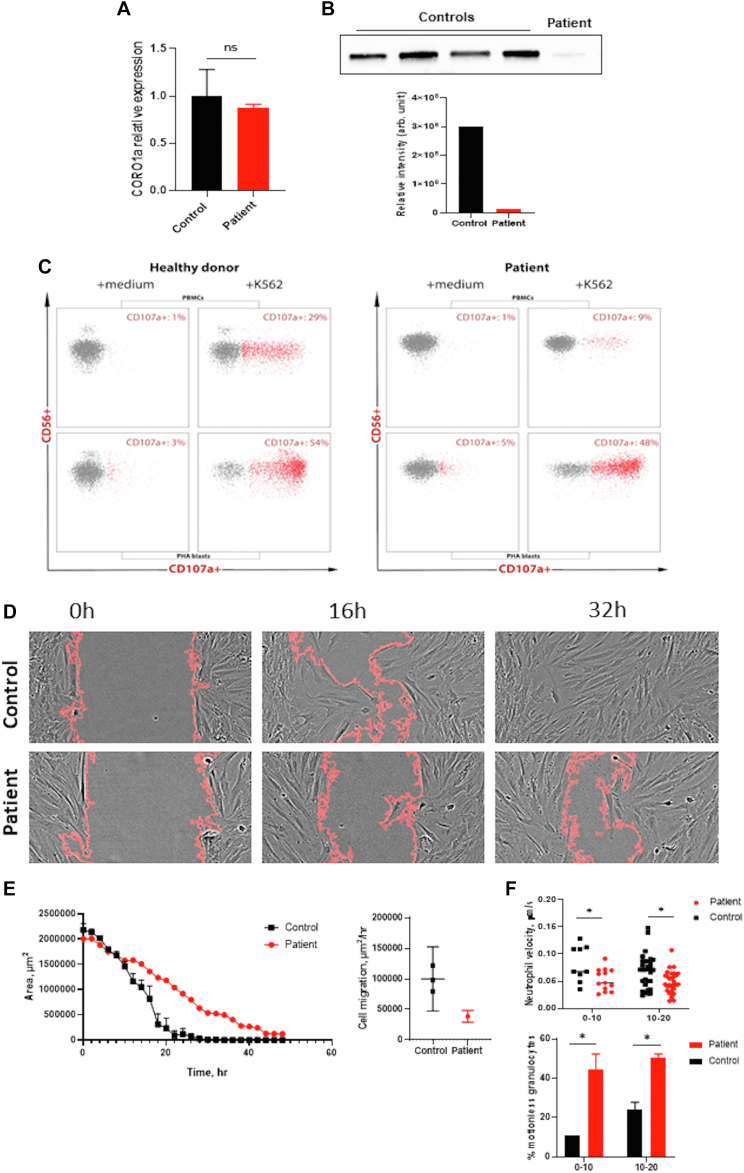
Impact of the detected gene variant on the CORO1A expression and primary cell functions. **A**, Real-time quantitative PCR analysis of CORO1a mRNA levels in the patient's MSC cells. Relative expression is reported as ΔΔCq normalized to glyceraldehyde-3-phosphate dehydrogenase (GAPDH) levels. Columns and bars represent means and SEMs, representative of 3 experiments. **B,** Immunoblot analysis of CORO1a levels in PBMC lysates from 4 control subjects and the patient before and after stem cell transplantation. Quantitative analysis of protein expression reported as a fluorescent signal normalized to total lane protein level. **C,** NK degranulation assay. **D,** Representative field of view of wound healing assay at the start, 16-hour, and 32-hour time points. **E,** Analysis of area change over time and cell front migration velocity during wound healing assay showing a significant attenuation in patients’ MSC cell levels and linear change in comparison with those of the healthy control. **F.** Velocity of the patient’s granulocyte crawling was diminished in comparison with that of the healthy donors, with an increased amount of motionless (average crawling velocity < 0.015 μm/s) granulocytes in the patient at all moments of time.

We attempted to verify the pathologic effects of the detected gene variant on CORO1A expression and primary cellular functions. The patient's COROA1 mRNA level was comparable to that of a healthy control; however, Western blot of her peripheral mononuclear cells revealed complete absence of the CORO1A protein (Fig 3, *A* and *B*).

Given that *CORO1A* is required for normal NK cells’ lytic function,^3^ we evaluated spontaneous NK cell degranulation, which proved to be decreased (9% vs the normal intralaboratory range of 18%- 60%) (Fig 3, *C*). The degranulation defect was moderate, as complete recovery of degranulation was observed after phytohemagglutinin/IL-2 stimulation (48% vs the normal range of 44%-77%).

We then performed a modified wound-healing assay with primary mesenchymal stem cells (MSCs) obtained from the patient’s bone marrow. The patient’s cells closed the defect significantly more slowly, requiring approximately 40 hours versus approximately 20 hours for the control (Fig 3, *D* and *E*). Interestingly, the MSCs also showed defects in cell motility despite generally significantly lower expression of CORO1A compared with that shown by hematopoietic cells. Recent studies have also shown that CORO1A plays an important role in fibroblast movement, not only via direct interaction with actin but also through several other mechanisms.^18^ Of note, our patient’s propensity for severe skin infections might be caused not only by systemic immunodeficiency but also by a local skin cellular dysfunction.

We also sought to assess the effect of protein loss on a different type of cellular motility, such as that seen in granulocytes. To assess their motility, we visualized granulocytes’ activity during thrombus formation in a flow chamber.^19^ The patient’s granulocyte crawling velocity was decreased in comparison with that of healthy donors, and the numbers of motionless or slowly crawling cells were increased (Fig 3, *F*). These data correspond with the described defects in mouse neutrophils deficient in CORO1A.^16^ Our findings demonstrate that CORO1A deficiency may lead to a combination of defects in cellular physiology and that in normal settings, CORO1A is required for neutrophil and MSC motility.

Finally, we explored the effects of the *CORO1A* mutation on platelet function. Calcium response to various stimuli was decreased in the patient’s platelets versus in those of normal controls; it was completely restored after HSCT (Table II). Similar calcium signaling defects were observed in CORO1A-deficient T lymphocytes.^20^ Interestingly, despite the attenuated calcium responses, our patient did not show any bleeding tendency.

**Table II:** Platelet intracellular signaling assay results in the patient versus in the healthy controls

Parameter	Activator	Patient before HSCT	Patient, after HSCT	Healthy donors
Cytosolic calcium level, nM	Resting	0.9	10.4	19 ± 15
	2 μM ADP	56.4	62.5	68 ± 38
	2 μg/mL of CRP	13.3	158.5	56 ± 30
	5 μM TRAP-6	92.9	166.5	180 ± 44
	100 μg/mL of fucoidan	32.0	15.5	10 ± 4
Fibrinogen binding, r.u.	Resting	0.5	0.45	3.3 ± 1.3
	2 μM ADP	4.9	16.73	8.2 ± 3.4
	2 μg/mL of CRP	1.8	5.69	2.1 ± 1.8
	5 μM TRAP-6	6.7	3.51	8.8 ± 3.7
	100 μg/mL of fucoidan	1.2	2.49	7.3 ± 6.5
Resting SSC, a.u.	Resting	4557.6	4278.9	7592 ± 1751
Change in SSC (%)	2 μM ADP	–21.8	–35.3	–30 ± 10
	2 μg/mL of CRP	–12	–31.2	–10 ± 5
	5 μM TRAP-6	–27	–35.5	–20 ± 10
	100 μg/mL of fucoidan	–25.3	–31.2	–40 ± 20
Annexin V–positive cells (%)	Resting	0.4	1.5	0.9 ± 0.3
	2 μM ADP	0.4	0.8	0.6 ± 0.3
	2 μg/mL of CRP	0.5	1.1	0.7 ± 0.4
	5 μM TRAP-6	0.3	0.8	0.7 ± 0.4
	100 μg/mL of fucoidan	1.4	1.2	0.6 ± 0.6

SSC flow cytometry represents platelet shape change after activation. Annexin V–positive cells are cells with phosphatidylserine exposure.

*ADP*, Adenosine diphosphate; *a.u.*, arbitrary unit; *r.u.*, relative unit; *SSC*, side scatter; *TRAP-6*, thrombin receptor activator for peptide 6.

Our functional experiments provide evidence of the damaging nature of the novel *CORO1A* variant. Interestingly, the heterozygous deletion of chromosome 16p11.2 (including the *CORO1A* gene) identified in our patient has been reported previously in 2 patients with hemizygous *CORO1A* mutations and the T^−^B^+^NK^+^ SCID phenotype with an early onset of symptoms, including severe life-threatening infections and EBV-associated diffuse large B-cell lymphoma.^1^^,^^7^ In contrast to these subjects, despite our patient’s low level of CORO1A expression, she had a milder immunologic phenotype: peripheral T cells that were decreased in number but present, a mildly decreased B-cell level, and quantitatively preserved NK cell compartments. The patient has experienced recurrent infections, yet she has not developed a malignancy, which is commonly reported in patients with CORO1A deficiency. This might be partially due to the EBV-negative status of the patients, as most malignancies described in CORO1A deficiency are EBV associated. Yet overall, our case observation suggests that the level of CORO1A protein expression may not be indicative of the disease’s severity. Although *CORO1A* mutations can lead to the development of SCID, it is also clear that alternative, milder phenotypes can develop from such mutations.

Like the 2 subjects who had *CORO1A* mutations in combination with chromosome 16 deletion, our patient also had behavioral impairment.^1^^,^^7^ CORO1A expression has been demonstrated in excitatory synapses, and the protein has been suggested to play an important role in cognition and behavior by regulating the cyclic AMP signaling pathway.^21^ Furthermore, CORO1A-deficient murine models have shown severe neurobehavioral defects, including defective socialization and increased aggression.^21^ Additionally, heterozygous loss of the genes in the 16p11.2 region might contribute to the neurobehavioral features in our patient, as people with similar deletions on chromosome 16 are known to have speech and/or developmental delay, attention deficiency disorder, epilepsy, and so forth (see the PubMed studies with the identifiers PMID 18184952, PMID 21940615, PMID 21731881, and PMID 19914906). Therefore, the underlying molecular mechanisms of these cognitive problems and their definitive association with CORO1A deficiency remain unclear and require further in-depth investigation.

The function of CORO1A in various cell types remains elusive. Our data indicate that the absence of CORO1A causes significant defects in platelet calcium signaling, degranulation defects in NK cells that are indicative of CORO1A role in synapse formation, and attenuated motility in primary neutrophils and mesenchymal stromal cells. These data suggest that CORO1A is crucial in actin cytoskeletal reorganization in most, if not all, hematopoietic cell lineages.

Assessing the direct role of the CORO1A protein in cytoskeletal reorganization in various cells remains a challenge. Recent studies have discussed CORO1A as a drug target for the treatment of multiple sclerosis, B-lineage leukemia, and other diseases, as well as a potentially emendable pathway by which to achieve long-term graft acceptance without compromising immunity to infections.^22^, ^23^, ^24^, ^25^ To avoid the side effects of such treatments, further studies exploring the molecular mechanisms involved are needed in animal and cellular models.

For detailed methods, please see the Methods section in this article’s Online Repository at www.jaci-global.org.

## Disclosure statement

Supported by a grant from the Foundation for Support and Development in the Field of Pediatric Hematology, Oncology and Immunology "Science for Сhildren" (Foundation "Science for Сhildren").

Disclosure of potential conflict of interest: The authors declare that they have no relevant conflicts of interest.

**Key messages**

• CORO1A defect significantly attenuates primary granulocytes’ mobility.
• CORO1A-deficient NK cells display decreased cytolytic function.
• CORO1A deficiency leads to a defect in primary mesenchymal stromal cells motility, possibly owing to abnormal cytoskeletal reorganization.

## Supplementary data

Online Repository
