# Supplementary material for: Novel hemizygous CORO1A variant leads to combined immunodeficiency with defective platelet calcium signaling and cell mobility
Source: J Allergy Clin Immunol Glob. 2023 Sep 27;3(1):100172. doi: 10.1016/j.jacig.2023.100172 (PMC10616384; doi:10.1016/j.jacig.2023.100172)
Supplement: Online Repository [file mmc1.docx]

**Online Repository**

**METHODS**

**Study subjects**

The studies involving human participants were reviewed and approved by the Independent Ethics Committee of Dmitriy Rogachev National Center of Pediatric Hematology, Oncology and Immunology (Moscow, Russia). After informed consent peripheral blood samples were drawn from the patient, and her parents and healthy adult voluntiers who served as a control.

**Genetic analysis and validation.**

Molecular genetic study was performed by next generation sequencing (NGS) method using target gene panel, containing 345 genes, associated with primary immunodeficiencies. DNA library preparation was performed by hybridization-based target enrichment method using custom probe panel, manufactured by Roche (Switzerland), following the manufacturer's protocol. DNA libraries were sequenced on an Illumina NextSeq (USA) using paired-end mode, according to the standard protocol for this platform. Average depth of target regions’ coverage was 130 reads per bp, 99.8% of bases – with target coverage at least 10x. Sequence reads were mapped to the human genome reference sequence (hg19). Single nucleotide variants (SNVs) and short insertions and deletions (INDELs) were identified using a proprietary bioinformatic data analysis pipeline, consistent with international standards. Pathogenicity and clinical significance of the identified variants was assessed by evaluating allele frequency, in silico predictions (SIFT, PolyPhen, PROVEAN, CADD, UMD Predictor, MutationTaster, etc.), annotations from public variant databases and related medical literature in accordance with the criteria of the American College of Medical Genetics and Genomics (ACMG) **[PMID: 25741868]**. Confirmation of the clinically relevant variants and genetic study of proband’s relatives was performed by direct Sanger sequencing on a capillary sequencer Genetic Analyzer 3500XL (Applied Biosystems, USA). Copy number was determined and visualized using ClinCNV package according to the developer recommendations. For detection of large-scale insertions and deletions was used exon-level comparative genomic hybridization on microchip (aCGH) with the Applied Biosystems CytoScan XON Array microchip, according to the manufacturer's protocol (Thermo Fisher Scientific Inc., USA).

The 16p11.2 deletion, detected in the patient, included the whole CORO1A gene, as well as 26 other genes: SMG1P2, SPN, QPRT, ZG16, KIF22, MAZ, PRRT2, PAGR1, MVP, CDIPT, CDIPTOSP, SEZ6L2, ASPHD1, KCTD13, TMEM219, TAOK2, HIRIP3, INO80E, DOC2A, FAM57B, ALDOA, PPP4C, TBX6, YPEL3, GDPD3, MAPK3. None of them are currently known to be associated with a disease in humans when carrying loss-off-function heterozygous defects.

**Immunological Assays**

The lymphocyte subsets were assessed using standard flow cytometry methods with the use of corresponding monoclonal antibodies (Becton Dickson, Franklin Lakes, NJ, USA) on the BD FACSCanto II (Becton Dickson). The levels of serum immunoglobulin were measured using the nephelometry technique on a BN ProSpec (Siemens, Berlin, Germany).

**Western Blot**

To obtain total protein lysates mononuclear cells were incubated with RIPA buffer (Sigma-Aldrich, USA) supplemented with Halt™ Protease and Phosphatase Inhibitor Single-Use Cocktail (Thermo Fisher Scientific, MA, USA) as recommended by the manufacturer. Total protein concentration was calculated using Pierce™ BCA Protein Assay Kit (Thermo Fisher Scientific, MA, USA). For western blot 10 ug of total protein were separated by SDS-PAGE on a Mini-PROTEAN TGX Stain-Free Precast Gels and transferred to a Immun-Blot Low Fluorescence PVDF Membrane (Bio-Rad Laboratories, USA) by wet blotting overnight. The membrane was washed with TBST twice and blocked with EveryBlot blocking buffer as recommended by manufacturer. The membrane was probed with Anti-Coronin 1a/TACO (ERP19467-36, Abcam, Cambridge, UK) recombinant monoclonal antibody at 1:1000 dilution overnight at 4 °C; after washing with TBST, membranes were incubated with 1:5000 goat anti-rabbit polyclonal antibodies conjugated with Alexa 647 Plus (Thermo Fisher Scientific, MA, USA) for 1 hour at room temperature. Membranes was visualized using ChemiDoc MP imaging system (Bio-Rad Laboratories, USA). Equal loading, transfer confirmations and total protein normalizations were performed using by Stain-Free membrane staining.

**Cell Culture**

Briefly, mononuclear cells were separated from bone marrow biopsy samples using Ficoll-Paque (GE-Healthcare, USA) according to manufacturer recommendations. Mesenchymal stem cells were then subsequently obtained from the mononuclear fraction by selecting adhering fibroblast-like cells for 20 days in α-MEM medium supplemented with 10% fetal bovine serum (Gibco, MA USA) and maintained at 37°C in 5% CO_2_.

**Wound Healing Assay**

Cell culture inserts in 6-well cell culture plates (Ibidi, Gräfelfing, Germany) were used to measure cell migration. Cell suspension in α-MEM medium was seeded at 7 × 10^5^ cells/ml in a two-well silicone insert with a defined cell-free gap and allowed to adhere for 24 hours at 37°C in 5% CO_2_. Before the assay the insert was removed to create a 500 µm gap and the well was filled with 2 mL of α-MEM medium supplemented with basic-FGF (Peprotech, UK) to promote cell migration. Images were obtained using IncuCyte SX1 (Sartorius, Göttingen, Germany) with 2 hours interval for 48 hours total at 37°C in 5% CO_2_. The obtained time-lapses were exported and analyzed using ImageJ.

**Quantitative Real-Time PCR**

RNA was isolated from cultured mesenchymal stem cells using AllPrep DNA/RNA/microRNA kit (Qiagen, Hilden, Germany). Isolated RNA was quantified using Qubit RNA BR Assay (Invitrogen, MA, USA) and assessed for purity using Nanodrop One (Thermo Fisher Scientific, MA, USA). First-strand cDNA was synthesized using LunaScript® RT SuperMix Kit (New England Biolabs, MA, USA). Primers were synthesized by Evrogen (Moscow, Russia). Primer sequences: CORO1a; Forward: CCAACATCGTCTACCTCTGTGG Revervse: CTCACACTTGTTCACCTCCAGG. GAPDH; Forward: GTCTCCTCTGACTTCAACAGCG, Reverse: ACCACCCTGTTGCTGTAGCCAA. Primer efficiency was determined beforehand and ~100% efficiency was achieved for every pair used. Real-time PCR was performed using the Luna® Universal qPCR Master Mix (New England Biolabs, MA, USA) with a manufacturer recommended protocol on a CFX 96 Real-time PCR system (Bio-Rad, USA) with triplicates for each sample. Results are reported as ∆∆Cq and analyzed with GraphPad Prism 8.0 (California, USA)

**Proliferation assay**

NK degranulation assay was performed using target cell line K562 as described previously. Data were acquired on a FACSCanto II (BD Biosciences) flow cytometer and analyzed using Diva Software 7.0 (BD Biosciences) and compared to the laboratory generated own reference values based on assays of 50 healthy donors.

**Tests for platelet functional activity**

Heterogeneity in platelet calcium signalling was assessed as described earlier [2], [3].

Continuous flow cytometry based assay of platelet intracellular signaling was performed as described previously [5,6].

Flow cytometry based platelet functional analysis was performed as described in detail in [7], [8].

**Granulocyte Crawling Assays**

Assessment of granulocytes activity during thrombus formation was performed as described earlier [9]. Thrombus growth and granulocyte crawling were visualized by means of Nikon Eclipse-Ti fluorescent microscope in low-angle mode. Granulocytes were identified from the thrombi by diffuse DiOC-6 staining and by the pattern of the cell movement.. Crawling granulocytes were identified and crawling velocities were quantified using Python 3.8.

**Detection of Mycobacterium species** was performed using DNA strip assay (GenoType Mycobacterium CM; Hain Lifescience, Germany) according to the manufacturer’s instruction.
